# Exploring the capability of mayenite (12CaO·7Al_2_O_3_) as hydrogen storage material

**DOI:** 10.1038/s41598-021-85540-8

**Published:** 2021-03-18

**Authors:** Heidy Visbal, Takuya Omura, Kohji Nagashima, Takanori Itoh, Tsukuru Ohwaki, Hideto Imai, Toru Ishigaki, Ayaka Maeno, Katsuaki Suzuki, Hironori Kaji, Kazuyuki Hirao

**Affiliations:** 1 Department of Materials Chemistry, Graduate School of Engineering, Kyoto University, Katsura A3-120, Nishikyo-ku, Kyoto, 615-8530 Japan; 2 Device Analysis Department, Nissan Arc, LTD., 1, Natsushima-cho, Yokosuka, Kanagawa 237-0061 Japan; 3 Frontier Research Center for Applied Atomic Science, Ibaraki University, 162-1 Shirakata, Tokai, Naka, Ibaraki 319-1106 Japan; 4 Institute for Chemical Research, Kyoto University, Uji, Kyoto 611-0011 Japan

**Keywords:** Energy science and technology, Engineering, Materials science

## Abstract

We utilized nanoporous mayenite (12CaO·7Al_2_O_3_), a cost-effective material, in the hydride state (H^−^) to explore the possibility of its use for hydrogen storage and transportation. Hydrogen desorption occurs by a simple reaction of mayenite with water, and the nanocage structure transforms into a calcium aluminate hydrate. This reaction enables easy desorption of H^−^ ions trapped in the structure, which could allow the use of this material in future portable applications. Additionally, this material is 100% recyclable because the cage structure can be recovered by heat treatment after hydrogen desorption. The presence of hydrogen molecules as H^−^ ions was confirmed by ^1^H-NMR, gas chromatography, and neutron diffraction analyses. We confirmed the hydrogen state stability inside the mayenite cage by the first-principles calculations to understand the adsorption mechanism and storage capacity and to provide a key for the use of mayenite as a portable hydrogen storage material. Further, we succeeded in introducing H^−^ directly from OH^−^ by a simple process compared with previous studies that used long treatment durations and required careful control of humidity and oxygen gas to form O^2^ species before the introduction of H^−^.

## Introduction

Mayenite (12CaO·7Al_2_O_3_) is a calcium aluminate compound and well-known constituent of high-alumina cement. Its unique emissive, optical, and chemical properties such as oxygen mobility^1–4^, ionic conductivity^5–8^, and catalytic performance^9–11^ have been explored and researched. The mayenite structure has the following stoichiometry: [Ca_24_Al_28_O_64_]^4+^ and anion sublattice 4X, with 12 crystallographic cages per unit cell (*I*4h3*d*) with *a* = 1.199 nm and a free space with a diameter of 0.4 nm^6^. The chemical and electrophysical properties of mayenite can be controlled by substitution of anions, such as O^−^, O^2−^, e^−^, OH^−^, H^−^, F^−^, and Cl^−^^12–18^. Although the introduction of hydride (H^−^) has been reported as a strategy to introduce electrons (e), there is no report on the possible application of mayenite in hydride state. We utilize the remarkable stability of hydrogen species inside mayenite and its affinity for water, which allows it to transform its cage nanostructure into a calcium aluminate hydrate, to explore the possibility of its use as a hydrogen carrier material.

Fossil fuels and natural gas are non-renewable and the generation of contaminants or non-environmentally friendly products from their combustion process poses a serious problem. Therefore, the demand for new alternate energy sources is increasing to resolve not only the environmental concerns but also the increase in the demand for fuels. Hydrogen is considered a great candidate for energy carriers to solve the aforementioned problems because it is a highly abundant, non-toxic, and renewable fuel^19–21^. In fuel cells, the only major oxidation product of hydrogen is water, with a minimal generation of harmful products compared with other energy sources. In addition, it contains a much larger chemical energy by mass (142 MJ) than any of the other hydrocarbon fuels. Moreover, it has energy by weight of 123 MJ kg^−1^, which is 3 times than that of gasoline and much higher than any of the other fossil fuels (e.g., diesel: 46 MJ kg ^−1^). However, its application is restricted due to delivery problems. Hydrogen is more of an energy carrier than an energy source^20,22–25^. Despite tremendous efforts to use hydrogen as a source of energy, a stable storage vehicle is still not easily accessible. Hydrogen storage is a key challenge in the development of hydrogen economy. Hydrogen can be stored in two physical forms, compressed gas and cryogenic liquid, so that it can be used as an energy source. However, storing hydrogen in these forms is complicated because of its low boiling point (− 252*.*87 °C) and low density in the gaseous state (0.08988 g/L) at 1 atm. Additionally, the transportation of high-pressure gas is not widespread because of safety risks and added costs. On the other hand, in the case of cryogenic systems, the low temperature requirements of insulated containers render the process very expensive^20,25^.

In particular, safe, cost-effective, and stable storage materials featuring efficient physical or chemical adsorption–desorption of hydrogen are needed for widespread applications of hydrogen, such as in portable electronics.

We propose the use of the nanocage structure of mayenite in the hydride state (H^−^) for the storage and safe transportation of hydrogen. Mayenite ceramics react with water, during which the ions trapped in the structure are easily desorbed. The easy desorption of hydride ions in water can allow the use of this material in portable applications. Additionally, this material is 100% recyclable because the cage structure can be recovered by the removal of water and subsequent heat treatment (1250 ℃ in air). We confirmed the presence of hydrogen as hydride by ^1^H-NMR spectroscopy, gas chromatography (GC), and neutron diffraction analyses. Further, we confirmed the hydrogen state stability inside the mayenite cage by first-principles calculations to better understand the adsorption mechanism and storage capacity and to provide a key to the development of mayenite as a hydrogen storage vehicle.

## Experimental

### Sample preparation

The mayenite 12CaO·7Al_2_O_3_ samples were prepared by the citrate gel technique using Ca(NO_3_)_2_・4H_2_O (Nacalai, 99.5%), Al(NO_3_)_3_・9H_2_O (Nacalai, 98.9%), and citric acid (C_6_H_8_O_7_) (Nacalai, 99%). The detailed preparation method is reported elsewhere^26^. Briefly, the citrate–nitrate was heated and stirred at 90 °C until a gel was formed and then heated for 2 h to evaporate excess water. The powder was then crushed and calcinated at 1250 °C for 3 h in air atmosphere. Thereafter, hydrogen treatment was conducted in a tubular furnace at 1250 °C for 2 h in a 100% hydrogen atmosphere. Additionally, we studied the effect of sintering time, hydrogen treatment time and temperature (for sintering at hydrogen treatment). We have reported the best conditions for hydrogen generation and omitted the details.

### Structure characterization

The crystal structures of the samples were analyzed using an X-ray diffractometer (XRD; Rigaku Corporation, RINT 2500HF) operated at 50 kV and 300 mA with a scanning rate of 0.02 s^−1^. The XRD analysis was carried out at room temperature, and Cu Kα radiation of 1.5406 Å wavelength was used. In addition, the diffraction angle (2*θ*) range was 10°–70°. The powder diffraction data were analyzed using JADE software to identify the phases present. The microstructures of the samples were analyzed using a field emission scanning electron microscope (FE-SEM; Nippon Electronics Co., Ltd., JSM-6705F) with an acceleration voltage of 3 kV. Neutron powder diffraction profiles were measured using a high-throughput diffractometer iMATERIA installed at the Japanese Particle Accelerator Research Complex (J-PARC). Rietveld refinements were performed using the program RIETAN-FP Version 2.32^27^ for XRD and Z-Rietveld Version 1.0.4.^28^, and 3D visualizer VESTA was used to demonstrate the crystal structures^29^.

### Cage characterization

^1^H-NMR spectroscopic measurements were performed using a Bruker AVANCE III 800 MHz US plus spectrometer equipped with a 2.5 mm MAS probe and operated at a resonance frequency of 800 MHz. Each sample was weighed to obtain quantitative results and sealed in a zirconia rotor. The MAS frequency was 30 kHz and the 1H 90 pulse length was 1.3 μs. Fully relaxed spectra were obtained with the recycle delay of 20 s. The chemical shifts were expressed as values relative to tetramethylsilane using the resonance line at 1.91 ppm for adamantane as an external reference.

Additionally, we measured ESR to verify the presence of O^2−^ in the cage structure.

### Hydrogen desorption

The desorption of hydrogen was verified by the reaction of the mayenite sample with water as follows: the sample (0.05 g) was added to distilled water (1 ml) at 60 °C in a head space recipient for 1 h. The sample was naturally cooled to room temperature and the gas inside the recipient was then analyzed by a gas chromatograph equipped with a thermal conductivity detector (GC-TCD) (GC–8A, Shimadzu Corporation) and a molecular sieve/5A column.

To determinate the activation energy from Arrhenius plot, we plotted desorption reaction temperature (RT ~ 80 °C) of dissolved mayenite in water versus the amount of hydrogen detected by the GC. The amount of hydrogen detected by the GC represented the desorbed hydride ions in the cage.

### Density functional theory calculation

DFT calculations were performed using OpenMx (an open-source package for Material eXplorer)^30^. The exchange correlation energy was approximated using the generalized gradient approximation^31^. An energy cutoff of 300 Ry was employed with a 2 × 2 × 2 k-point grid in the 124 and 122 atoms unit cell for structural optimization. We used the following base functions: *s*4*p*3*d*3 for Ca, *s*3*p*3*d*2 for Al, *s*2*p*2*d*1 for O, and *s*2*p*1 for H. The cutoff radii were chosen as 11.0, 8.0, 6.0, and 6.0 au for Ca, Al, O, and H, respectively. The convergence criteria were set to 2.0 × 10^−4^ Hartree/Bohr or 1.0 × 10^−5^ Hartree for structural optimization. The structures are visualized using Materials Studio Visualizer 8.0^32^.

## Results and discussion

### Structure and cage characterization

**Figure 1:**
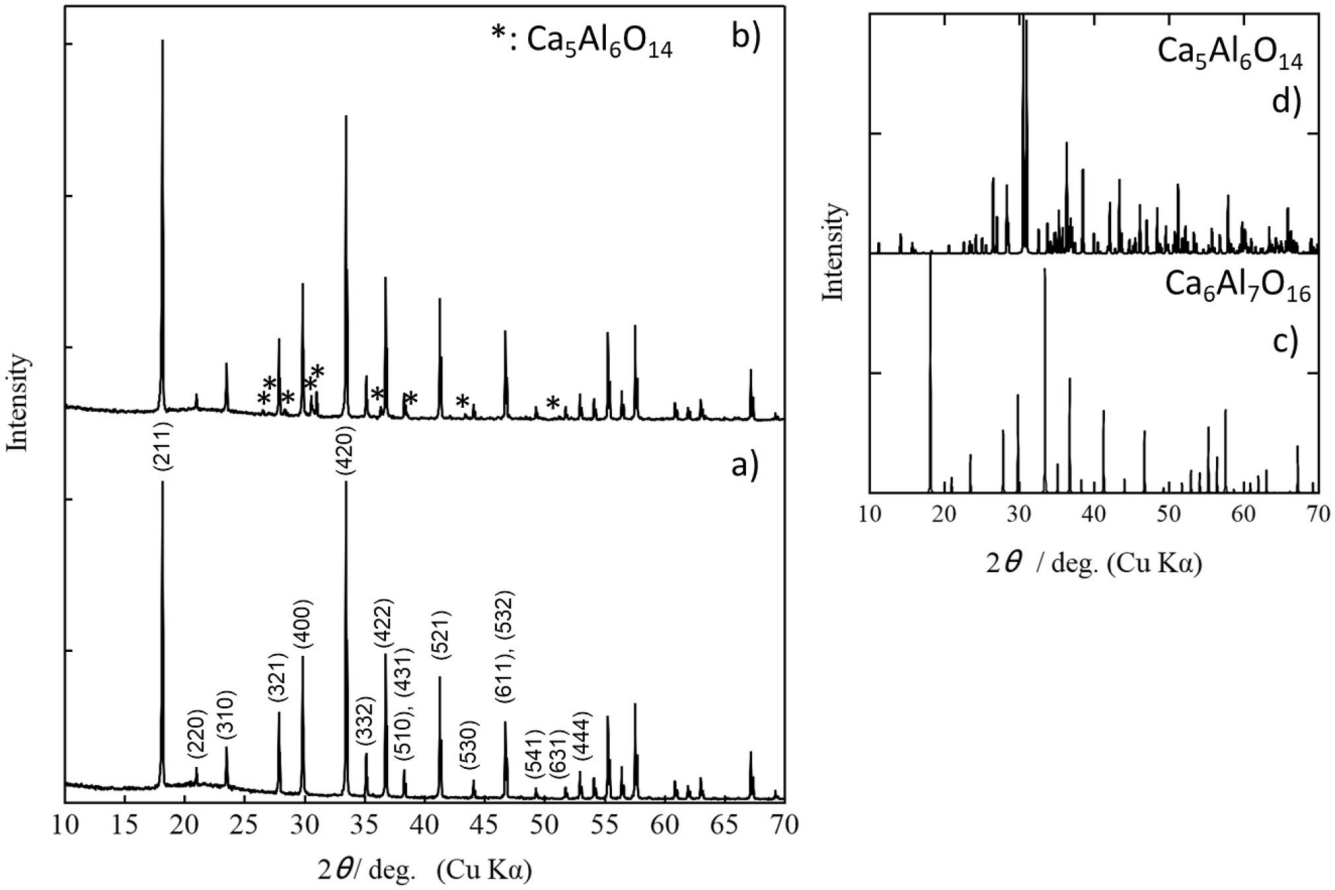
XRD patterns of mayenite (Ca_12_Al_14_O_33_). (**a**) XRD pattern before hydrogen treatment and (**b**) XRD pattern after hydrogen treatment. (**c**) Ca_6_Al_7_O_16_ and (**d**) Ca_5_Al_6_O_14_ simulated XRD patterns.

**Figure 2:**
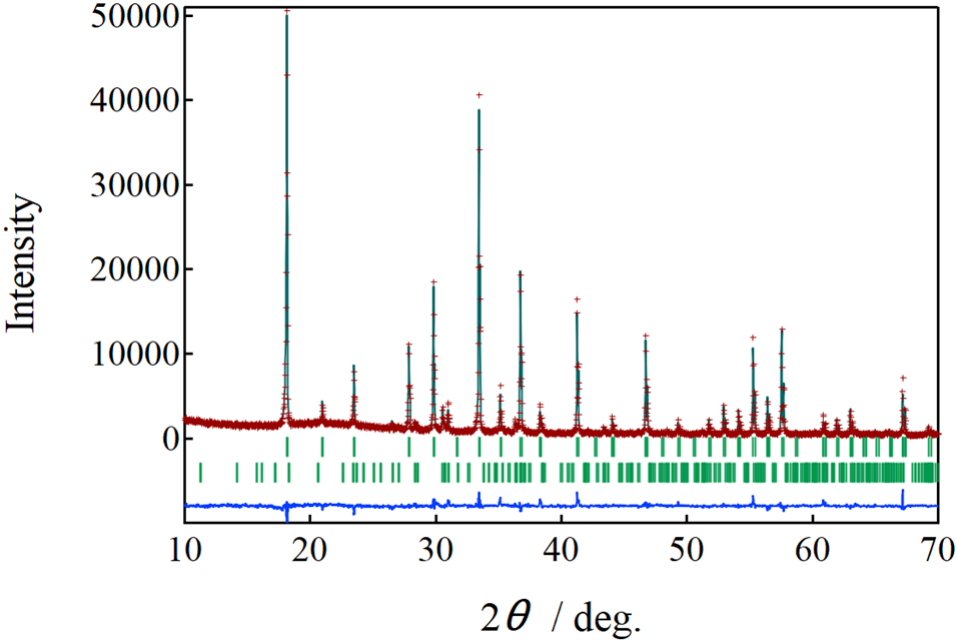
Rietveld refinement profile of X-ray diffraction of Ca_6_Al_7_O_16_ and Ca_5_Al_6_O_14_. Observed (brown crosses), calculated (green line), Ca_6_Al_7_O_16_ Bragg position (1^st^ green perpendicular line), Ca_5_Al_6_O_14_ Bragg position (2nd green perpendicular line), and difference between observed and calculated (lowest blue line).

Figure 1a, b show the XRD patterns of the mayenite samples before and after hydrogen treatment. The XRD profile of the mayenite sample prepared before H_2_ gas treatment agrees well with that of the Ca_6_Al_7_O_16_ structure (Inorganic Crystal Structure Database (ICSD) No. 241241) having a cubic system with *I*43*d* space group (No. 220). On the other hand, the XRD profile of the sample treated with H_2_ gas indicates a main phase composed of Ca_6_Al_7_O_16_ structure (ICSD No. 241241) and sub-phase composed of Ca_5_Al_6_O_14_ structure (ICSD No. 1714) having an orthorhombic system with *Cmc*2_1_ space group (No. 36). Figure 1c, d show XRD patterns of Ca_6_Al_7_O_16_ and Ca_5_Al_6_O_14_ phases simulated by RIETAN-FP^27^, respectively. The content of Ca_5_Al_6_O_14_ phase is approximately 5 wt% estimated by Rietveld analysis in Fig. 2.

**Figure 3:**
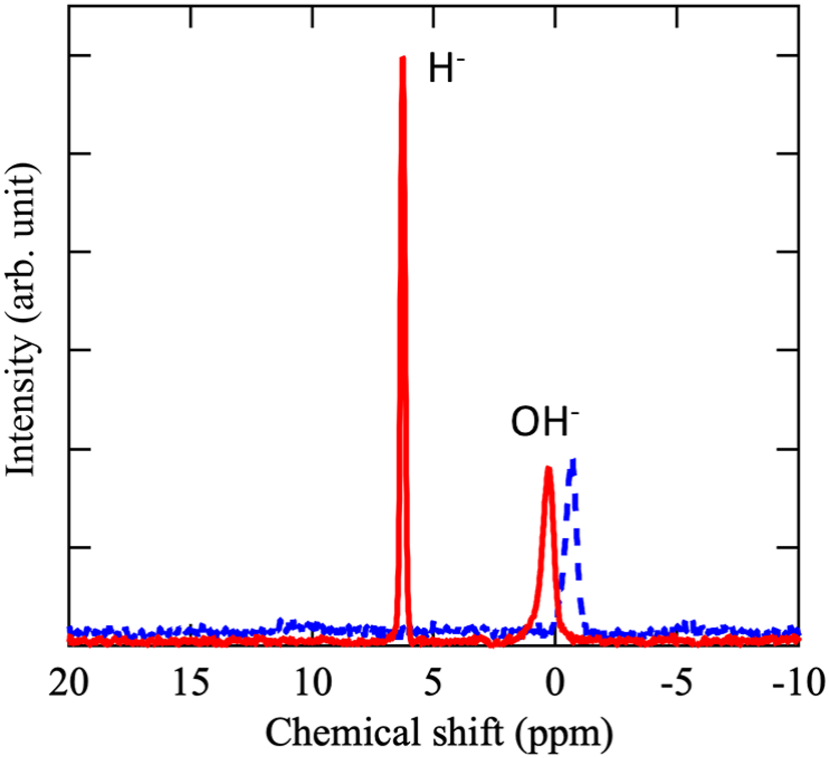
Solid-state ^1^H magic-angle-spinning NMR spectra of Ca_6_Al_7_O_16_. Dashed line: NMR spectrum before H_2_ treatment. Continuous line: NMR spectrum after H_2_ treatment.

Solid-state ^1^H magic-angle-spinning (MAS) NMR was used to analyze the presence of H^−^ or OH^−^ inside the cage and their amounts before and after hydrogen treatment. The NMR results are shown in Fig. 3. The results show the presence of two peaks for the all the analyzed samples after hydrogen treatment. The peak around 6.1 ppm corresponds to H^−^ and that at − 0.75 ppm corresponds to OH^−^. The assignment was carried out based on a previous study^33^.

It further, the NMR results suggest that a part of H^−^ was introduced into the cage, while OH^−^ remained on the sample despite hydrogen treatment. Assuming that 4 ions can be introduced in the free cages, we calculated the fraction amount of OH^−^ and H^−^ from the NMR data. The calculations were based on the integration of the peak area for H^−^ and OH^−^. Based on these fractions, the amount of H^−^ inside the cage was calculated to be 7.3 × 10^–4^ mol g^−1^. This corresponds to 17.9 ml of hydrogen by grams of mayenite.

**Figure 4:**
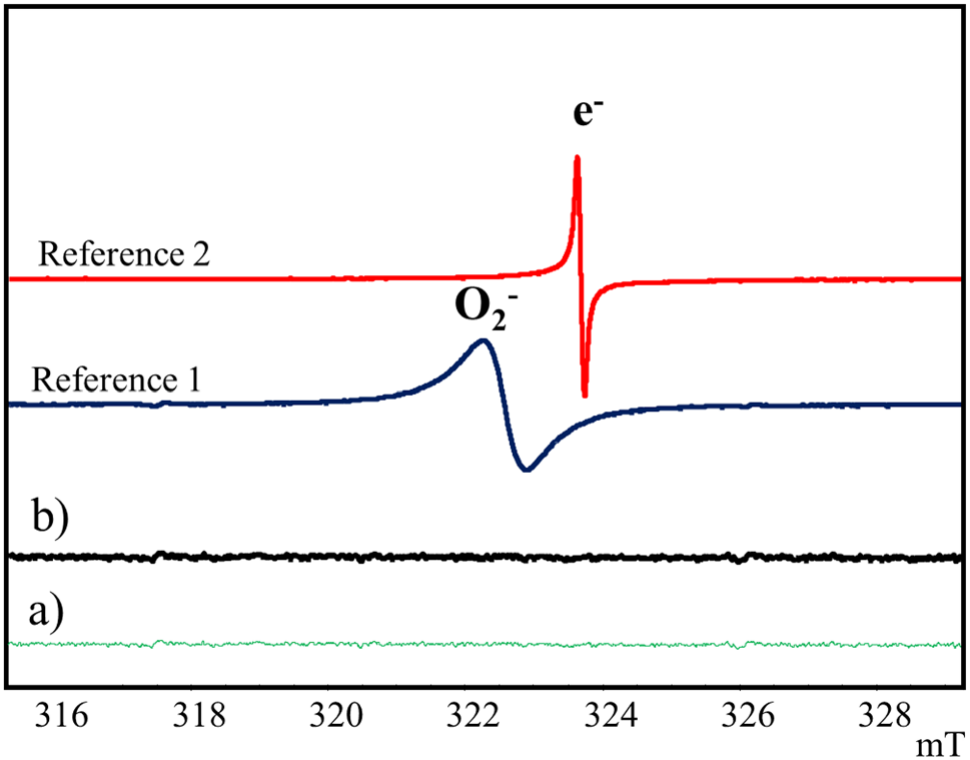
ESR spectra of mayenite (Ca_12_Al_14_O_33_). (**a**) NMR spectrum before hydrogen treatment and (**b**) NMR spectrum after hydrogen treatment.

ESR analysis, shown in Fig. 4 did not show any presence of ESR signals due to O^2−^ (*g*_*z*_ = 2.020) in the structure. In Fig. 4, the ESR spectra of O^2−^ and e^−^ are shown as reference spectra.

**Figure 5:**
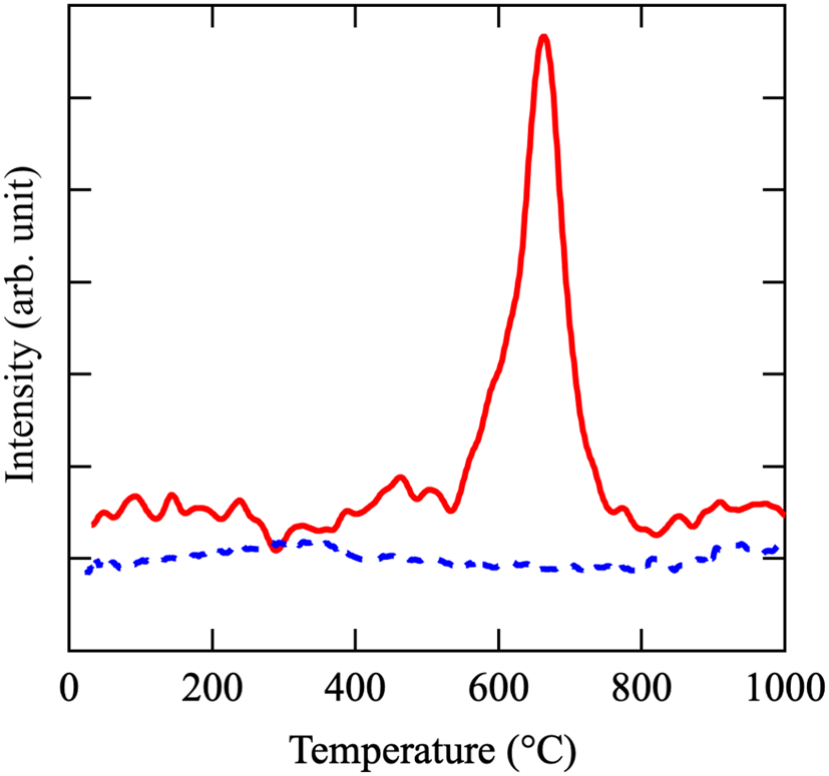
Thermogravimetry–differential thermal analysis–photoionization mass spectra for Ca_6_Al_7_O_16_. Continuous line: After treatment with H_2_ gas. Dashed line: Before treatment with H_2_ gas.

Additionally, to verify the hydrogen state and possible adsorption on the surface, we analyzed the sample after hydrogen treatment by thermogravimetry differential thermal analysis photoionization mass spectrometry (TG–DTA–PIMS). The DTA–TG–PIMS data were collected under He flow and the result is presented in Fig. 5. The observed temperature versus gas evolution profile of the mayenite sample hydrogen treated at 1250 °C for 2 h shows a strong evolution peak of H_2_ centered at approximately 600 °C (from IC *m/z* = 2 band of MS). These results indicate that hydrogen was not present at the surface and all the hydrogen was stored in the cage. Additionally, Fig. 5 shows that the H_2_ peak is absent for the sample before hydrogen treatment.

### Crystallography

**Figure 6:**
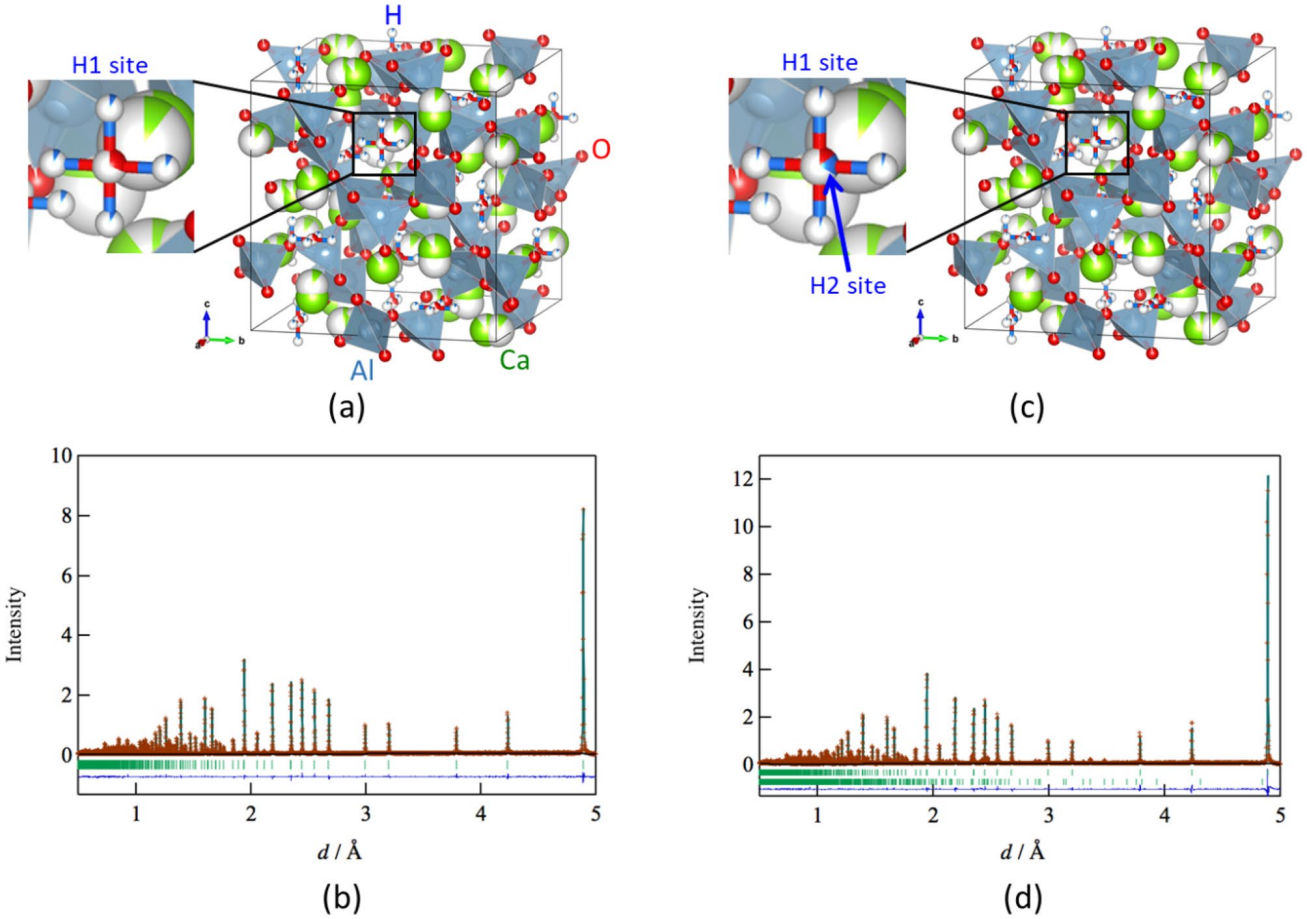
Lattice frameworks and Rietveld refinement profiles of time-of-flight neutron diffraction ofCa_6_Al_7_O_16_. (**a**) Lattice frameworks of (**a**) as-sintered Ca_6_Al_7_O_16_ and (**c**) H_2_ gas-treated Ca_6_Al_7_O_16_. Rietveld refinement profiles of time-of-flight neutron diffraction of (**b**) as-sintered Ca_6_Al_7_O_16_ and (**d**) H_2_ gas-treated Ca_6_Al_7_O_16_.

Hayashi et al.^33^ studied the information of H^−^ in Ca_6_Al_7_O_16_ by the Rietveld and Maximum Entropy Method analyses using synchrotron X-ray diffraction data. However, X-rays are scattered by H atom and H^−^ ions. Therefore, we estimated the structural parameters of H atom and H^−^ ion in Ca_6_Al_7_O_16_ with and without H_2_ treatment by the Rietveld analysis using time-of-flight (TOF) neutron diffraction data. The cubic model given in Supplementary Table S1 in the supporting information was used for the Rietveld analysis. Figure 6a–d show the crystal structures and the results of the Rietveld analysis of Ca_6_Al_7_O_16_ without and with H_2_ treatment. Supplementary Tables S2 and S3 list the structural parameters and reliability factors (R factors) of the non-H_2_-treated and H_2_-treated samples. The R factors of these samples obtained from the Rietveld analysis are satisfactory for the discussion of structural parameters. Most of the structural parameters such as lattice parameter, coordinate fraction, and bond length are not different for the non-H_2_-treated and H_2_-treated samples. The Ca 24d site was the split-site in non-H_2_ and H_2_-treated samples. The oxide ion of the O–H ions in the non-H_2_ treated sample is located at the 12a site in the Ca_6_Al_7_ cage. The protons of the O–H ions in the non-H_2_ and H_2_-treated samples are expected to be located at the 48e site in the Ca_6_Al_7_ cage; however, they are not localized at the 48e site because of the large atomic displacement parameter estimated by Rietveld analysis. Further, the H atoms and/or H^−^ ions in the non-H_2_-treated sample were not located at the 12a site in the Ca_6_Al_7_ cage, whereas in the H_2_-treated sample, they were located at the 12a site in the Ca_6_Al_7_ cage. The occupancy of the 12a site for the H atom was approximately 0.2 in the H_2_-treated sample with a decrease in the occupancy of the 12a site for the oxide ion. However, the Rietveld analysis using the TOF neutron diffraction data was unable to define the H atoms and/or H^−^ ions at the 12a site in the Ca_6_Al_7_ cage. Therefore, we attempted to estimate the stability of the H atoms and H^−^ ions at the 12a site in the Ca_6_Al_7_ cage by density functional theory (DFT) calculations.

### Extraction mechanism

#### Hydrogen desorption

**Figure 7:**
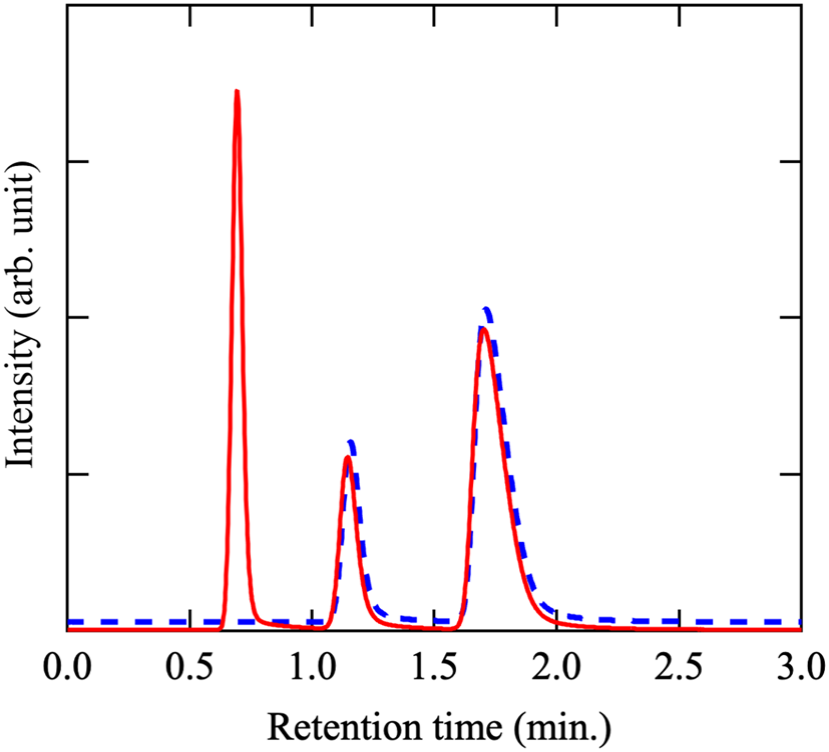
Gas chromatography–thermal conductivity detector spectra for hydrogen desorption from Ca_6_Al_7_O_16_. Continuous line: After treatment with H_2_ gas. Dashed line: Before treatment with H_2_ gas.

The storage hydrogen amount was confirmed using the GC-TCD. Figure 7 shows the GC-TCD results for mayenite ceramics before and after hydrogen treatment, in pure water at 60 °C. Retention time of 0.58, 1.25, and 1.8 min corresponds to hydrogen, oxygen, and nitrogen gases, respectively. The sample before hydrogen treatment only showed the peaks corresponding to oxygen and nitrogen. Oxygen and nitrogen peaks originate due to the presence of air in the head space.

The amount detected from GC results is 18.14 ml of hydrogen per gram of mayenite. These results are in good agreement with the theoretical amount (17.9 ml g^−1^) calculated from the NMR results, which is listed in Table 1.

**Table 1:** Hydrogen amount estimated by NMR and GC-TCD analyses.

NMR (ml/g)	GC-TCD (ml/g)
17.9	18.14

However, these results correspond to a storage density of < 1 mass%, which is a very low energy density to be useful for real-world applications. Further studies are needed to improve the amount of H^−^ in the cage to use mayenite as a possible hydrogen storage material.

**Figure 8:**
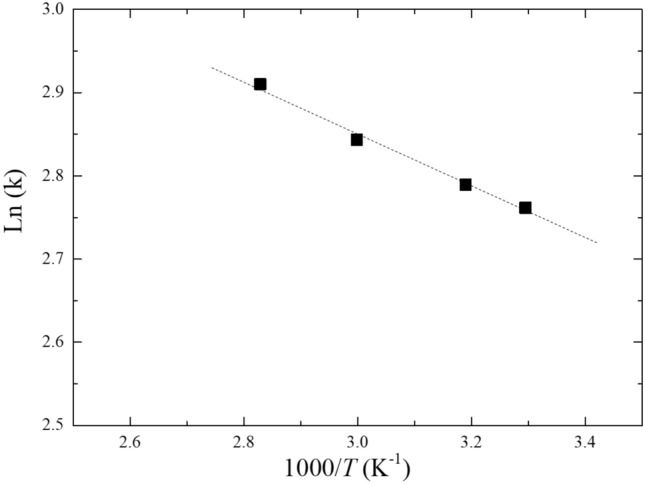
Arrhenius plot of the hydrogen amount released by mayenite in water.

The mayenite, 12CaO·7Al_2_O_3,_ samples completely decomposed in water. Therefore, the hydride species trapped inside the cage were released and generated hydrogen. This reaction was almost independent of temperature as shown by Fig. 8 (Arrhenius plot). The activation energy of hydrogen released from mayenite cage in water was calculated to be 2.6 kJ mol^−1^ from the slope of the line in the graph by using the following equation:

$${\text{Ln}}\left( k \right) = \, - 315.77\left( {1/T} \right) \, + \, 3.7981$$

This very low value of activation energy is because almost no energy is required to dissolve mayenite in water.

Mayenite with H^−^ ions dissolves in water according to the following reaction.

1 $$\left[ {{\text{Ca}}_{24} {\text{Al}}_{28} {\text{O}}_{64} } \right]^{4 + } \cdot 4H^{ - } + 50{\text{H}}_{2} {\text{O}} \to 8{\text{Ca}}_{3} {\text{Al}}_{2} \left( {{\text{OH}}} \right)_{12} + 6{\text{Al}}_{2} {\text{O}}_{3} + 4{\text{H}}_{2}$$

The cage structure can be recovered by removal of water and then applying heat treatment (1250 ℃ in air as follows:

2 $${\text{Ca}}_{3} {\text{Al}}_{2} \left( {{\text{OH}}} \right)_{12} \to {\text{Ca}}_{3} {\text{Al}}_{2} {\text{O}}_{6} + 6{\text{H}}_{2} {\text{O}}$$

3 $$4{\text{Ca}}_{3} {\text{Al}}_{2} {\text{O}}_{6} + 3{\text{Al}}_{2} {\text{O}}_{3} \to \left[ {{\text{Ca}}_{24} {\text{Al}}_{28} {\text{O}}_{64} } \right]^{4 + } \cdot 4{\text{OH}}^{ - }$$

**Figure 9:**
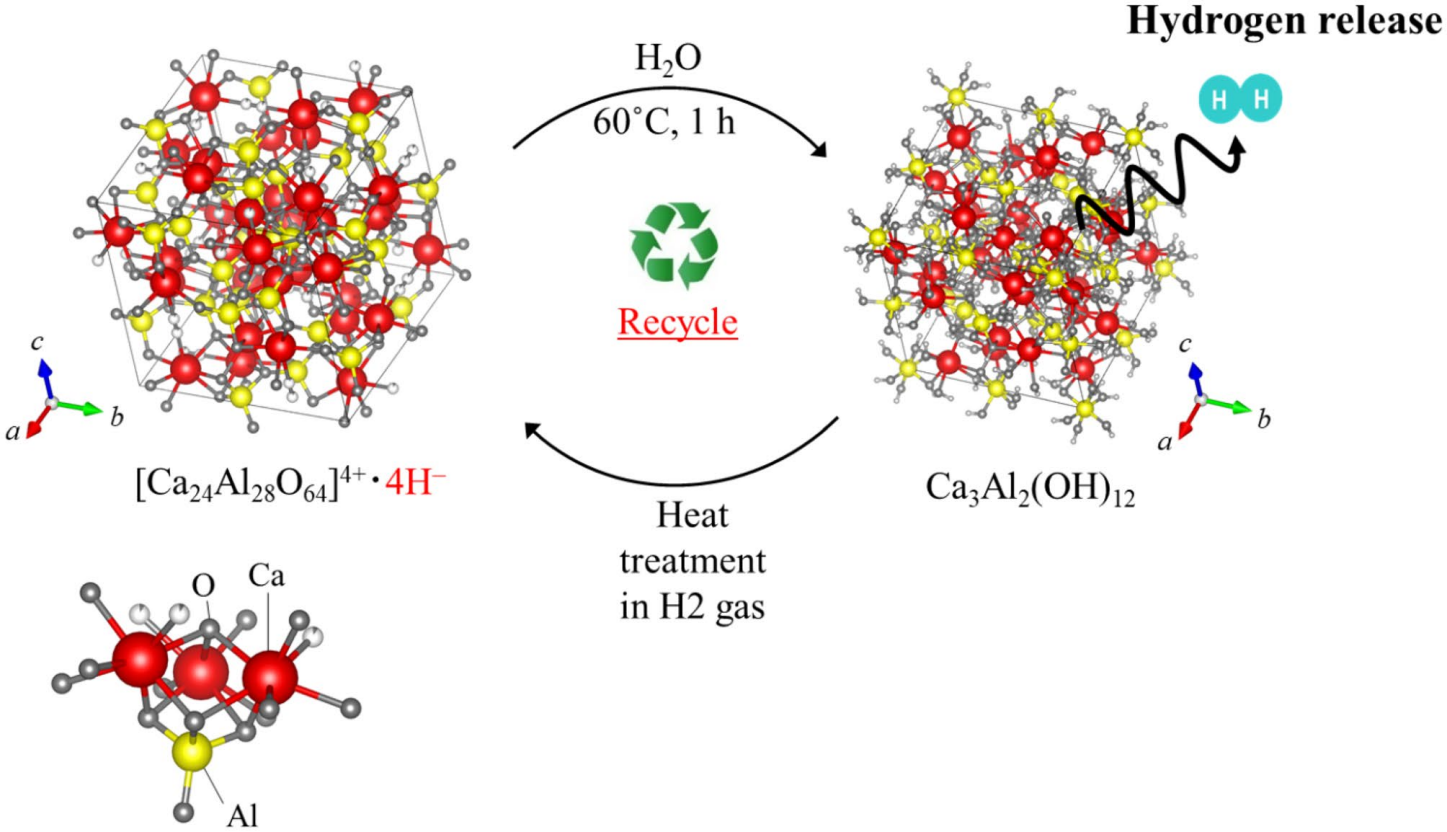
Schematic depicting recycling of mayenite as a possible solid-state hydrogen storage material.

A schematic representation of the possible cycle life of mayenite is shown in Fig. 9. After dissolution in water and hydrogen release, mayenite can be recovered completely (100%) by 2 h heat treatment in air at 1250℃. We verified the structure by XRD analysis and the hydrogen treatment was performed. The amount of hydrogen released was verified by GC-TDC. The results are showed in S1.

#### Storage mechanism

The mechanism of H^−^ formation in mayenite has been discussed in a previous study^34^. Here, we briefly discuss the mechanism from the viewpoint of comparison with our experiments. After calcination in air, we confirmed the structure of mayenite by NMR as [Ca_24_Al_28_O_64_]^4^·4OH^−^, as explained in the previous section (cage characterization part).

Mainly two types of hydrogen doping mechanisms have been reported^35^. The first is the adsorption of a H_2_ molecule from the gas phase onto the mayenite surface with subsequent dissociation into a pair of either H^0^ or H^+^ and H^−^ ions. Then, the H atoms or ions diffuse into the bulk with a concentration gradient. This process involves long treatment times for the hydrogen to diffuse and dissociate^2,3,13^. However, in this study, the annealing duration in hydrogen atmosphere is very short (2 h) compared to the annealing duration (> 24 h)^2,3,13^ reported in literature. Therefore, it is hard to assume this mechanism as a possible route for the H^−^ formation in the cage.

Another proposed mechanism is the diffusion of H_2_ molecules into the mayenite bulk and their participation in the chemical reaction^34^. This mechanism if more feasible in this study, assuming that H_2_ rapidly diffuses into the cages of mayenite and then undergoes chemical reactions with OH^−^ inside the cage as follows:

2 $${\text{OH}}^{ - }_{{({\text{cage}})}} + {\text{H}}_{{2({\text{g}})}} \to {\text{H}}^{ - }_{{({\text{cage}})}} + {\text{H}}_{2} {\text{O}}$$

Another possibility is that at elevated temperatures, dehydroxylation of the surface forms O^2−^ surface sites followed by exchange with H^−^ ions as represented by the following equation:

3 $${\text{O}}^{2 - } + 2{\text{H}}_{2} \to 2{\text{H}}^{ - } + {\text{H}}_{2} {\text{O}}$$

However, as discussed previously, O^2−^ was not present our samples, which was confirmed by the ESR results. To introduce O^2−^ in the cage, the sintering environment should be carefully controlled (generally, control of humidity in the oxygen atmosphere)^2,3,13^^.^ However, our experiments were simplified to explore the possibility of using mayenite as a hydrogen carrier in real-world applications. Thus, we sintered the samples in air without controlling the humidity and/or oxygen gas atmosphere. This was the reason for the absence of O^2−^ in our samples.

#### Density functional theory calculations

**Figure 10:**
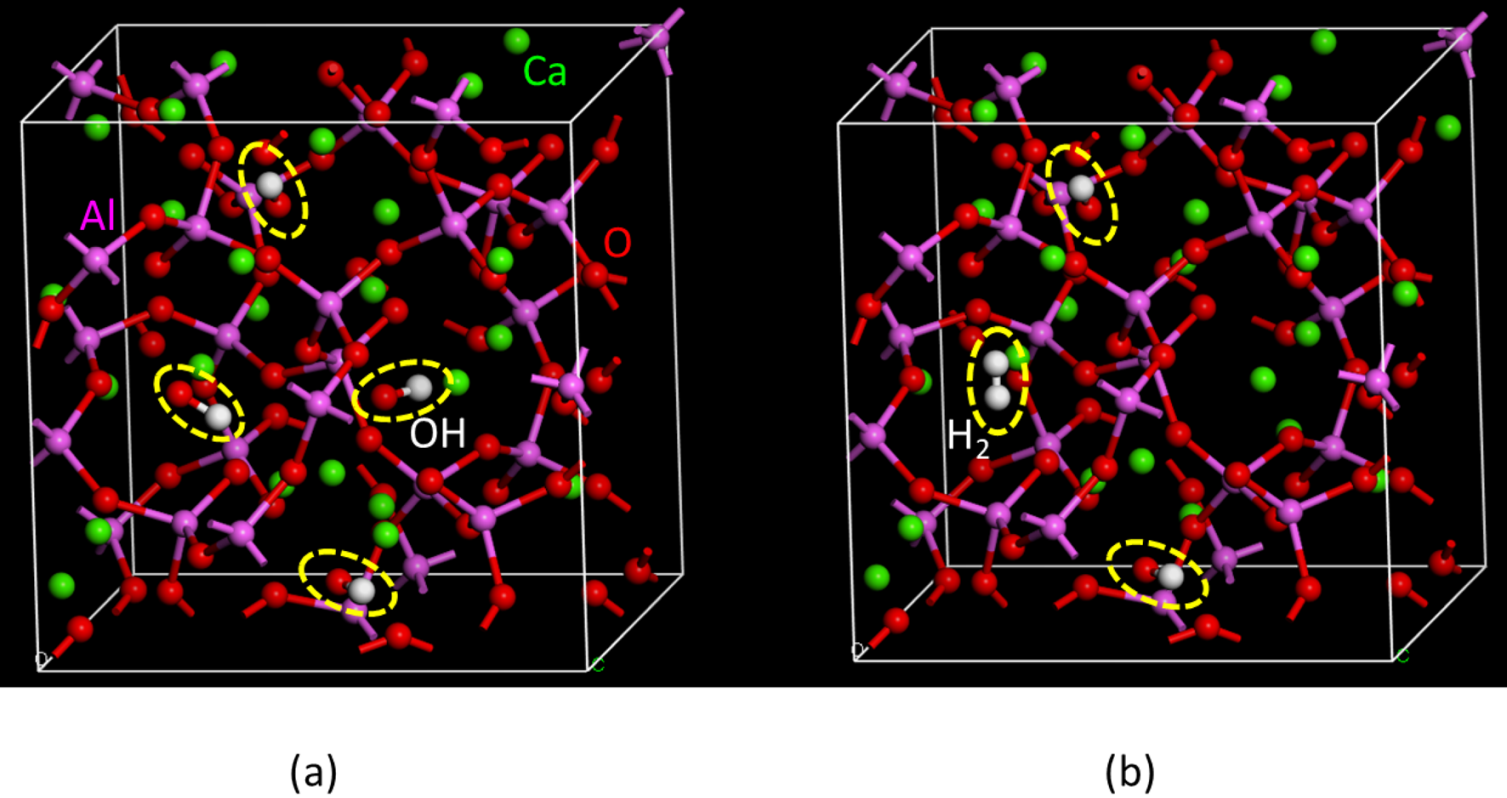
Optimized structures by density functional theory calculation. (**a**) Ca_24_Al_28_O_64_ + 4OH model and (**b**) Ca_24_Al_28_O_64_ + 2OH + H_2_ model.

**Figure 11:**
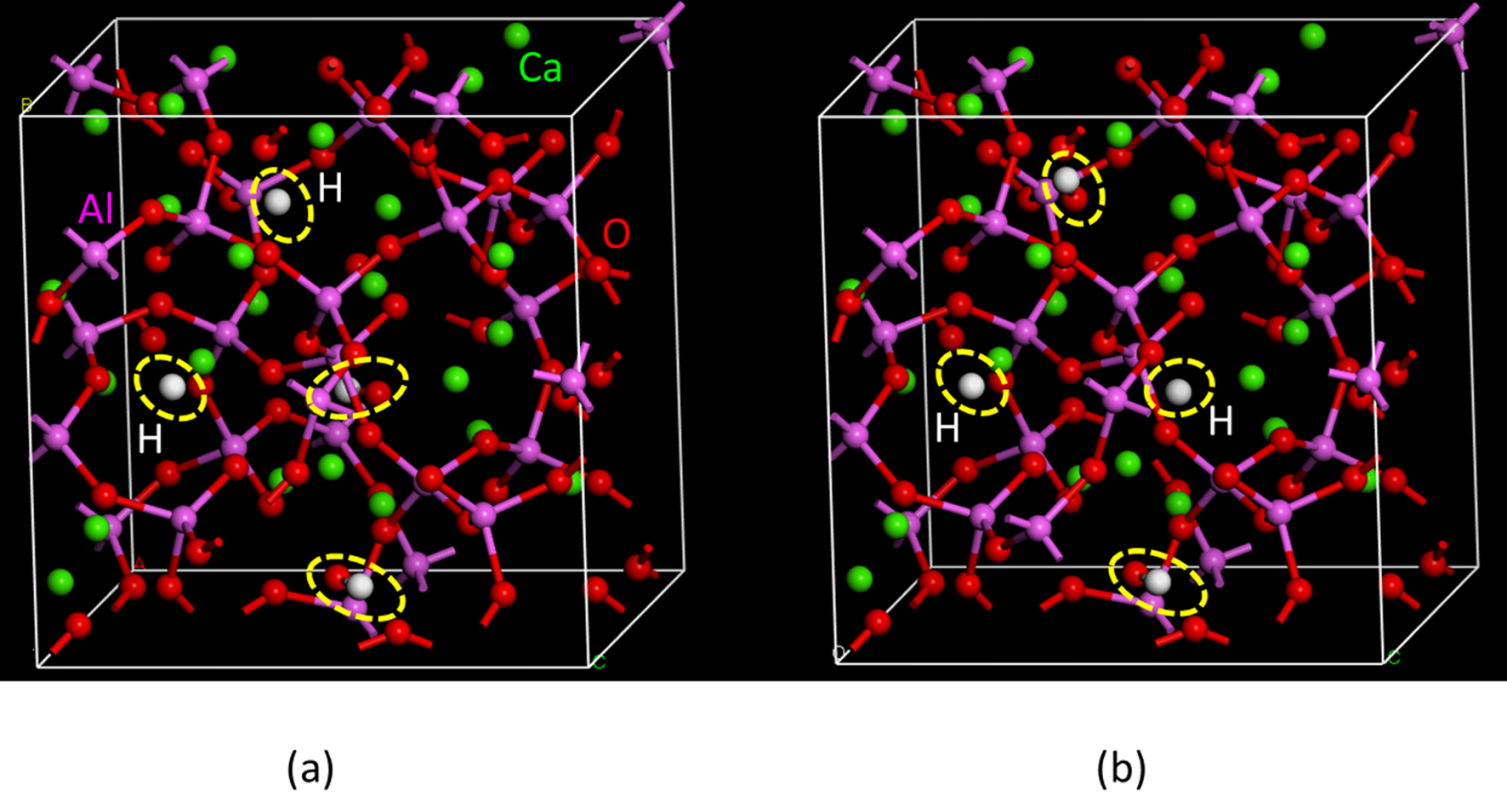
Optimized structures by density functional theory calculation for Ca_24_Al_28_O_64_ + 2OH + 2H model. (**a**) Each H exists in adjacent site and (**b**) each H exists in non-adjacent site.

We studied the hydrogen states, viz., H^+^, H^−^, and/or H_2_, in the mayenite treated by H_2_ gas using the DFT calculations. The calculated models of the crystal structures with P 1 obtained from the results of the Rietveld analysis, Ca_24_Al_28_O_64_ + 4OH and Ca_24_Al_28_O_64_ + 2OH + 2H, were optimized and evaluated under the constraints of lattice parameters, viz., a = b = c and α = β = γ = 90° (Figs. 10, 11). The structure defined by the Rietveld analysis using the TOF neutron diffractions were used for the initial structure model in DFT calculations, as shown in Tables S2 and S3. In this study, we performed DFT calculations for three models: Ca_24_Al_28_O_64_ + 4OH, Ca_24_Al_28_O_64_ + 2OH + H_2_, and Ca_24_Al_28_O_64_ + 2OH + 2H. No significant differences were observed in the Ca_24_Al_28_O_64_ structures with and without H and/or H_2_ in the base structure.

In the optimized structure of the Ca_24_Al_28_O_64_ + 2OH + H_2_ model, the H–H bond length in H_2_ was approximately 1.759 Å, which is considerably longer than the general H–H length in H_2_. The Ca_24_Al_28_O_64_ + 2OH + 2H model was approximately 1.34 eV more stable than the Ca_24_Al_28_O_64_ + 2OH + H_2_ model. The DFT calculation results confirm the validity of the Ca_24_Al_28_O_64_ + 2OH + H_2_ model. The H existing in the adjacent site model of Ca_24_Al_28_O_64_ was approximately 3 meV more stable than the H existing in the non-adjacent site model of Ca_24_Al_28_O_64_. However, the energy difference between the two models was found to be very small. We evaluated the average charge of each element using the Mulliken analysis, which is listed in Table 2. It can be seen that both H and H_2_ were negatively charged in Ca_24_Al_28_O_64_. The analysis results agree with the NMR results. From both experiments and DFT calculations, we concluded that hydrogen exists as H^−^.

**Table 2:** Average charge density (Mulliken charge) of atoms in Ca_6_Al_7_O_16_ estimated by density functional theory calculations.

Atom	Ca_24_Al_28_O_64_ + 4OH	Ca_24_Al_28_O_64_ + 2OH + H_2_	Ca_24_Al_28_O_64_ + 2OH + 2H
Al	+ 0.632	+ 0.635	+ 0.654
Al_d_	+ 0.560	+ 0.565	+ 0.581
O	− 0.550	− 0.550	− 0.549
O_d_	− 0.566	− 0.566	− 0.562
Ca	+ 0.875	+ 0.849	+ 0.810
OH	− 0.526	− 0.523	–
H_2_	–	− 0.219	–
H	–	–	− 0.254

Al*d* Al atom bonded to O atom with dangling bond, O*d* O atom with dangling bond.

## Conclusions

In summary, we successfully demonstrated the application of mayenite (12CaO·7Al_2_O_3_ ceramic) as a potential hydrogen storage material, There are no requirements of high temperatures or pressures for desorption, because mayenite has the advantage of hydrogen desorption by dissolution of mayenite in water through a reaction at a relatively low temperature (60 °C at 1 h). After the reaction with water, the cage structure of mayenite is transformed into a calcium aluminate hydrate and this transformation enables hydrogen desorption at a low temperature. The mayenite can be recovered by applying heat treatment to calcium aluminate hydrate and the subproducts generated in the reaction with water. The activation energy for hydrogen desorption in water was calculated to be 2.6 kJ mol^−1^. Additionally, this material is highly stable in air and water vapor environments at low temperatures^33^, which is an advantage for its possible use as hydrogen carrier. However, the energy density is very low (less than 1 mass%) to be useful in real-world applications. There is a need to improve the amount of hydride adsorption sites in mayenite by surface treatments or other techniques.

## Supplementary information

Supplementary information.
