# Supplementary material for: Exploring the capability of mayenite (12CaO·7Al2O3) as hydrogen storage material
Source: Sci Rep. 2021 Mar 18;11:6278. doi: 10.1038/s41598-021-85540-8 (PMC7973484; doi:10.1038/s41598-021-85540-8)
Supplement: Supplementary file 1 — Supplementary information. [file 41598_2021_85540_MOESM1_ESM.docx]

Supplementary Information

------------------------------------------------------------------------------------

Exploring the capability of mayenite (12CaO·7Al_2_O_3_) as hydrogen storage material

Heidy Visbal^1^, Takuya Omura^1^, Kohji Nagashima^1^, Takanori Itoh^2^, Tsukuru Ohwaki^2^, Hideto Imai^2^, Toru Ishigaki^3^, Ayaka Maeno^4^, Katsuaki Suzuki^4^, Hironori Kaji^4^, Kazuyuki Hirao^1^*

^1^ Department of Materials Chemistry, Graduate School of Engineering, Kyoto University, Katsura, Nishikyo-ku, Kyoto 615-8530, Japan

^2^ Device Analysis Department, Nissan Arc, LTD., 1, Natsushima-cho, Yokosuka, Kanagawa 237-0061, Japan

^3^ Frontier Research Center for Applied Atomic Science, Ibaraki University, 162-1 Shirakata, Tokai, Naka, Ibaraki 319-1106, Japan

^4^ Institute for Chemical Research, Kyoto University, Uji, Kyoto 611-0011, Japan

*Corresponding Author: [hirao@bisco1.kuic.kyoto-u.ac.jp](mailto:hirao@bisco1.kuic.kyoto-u.ac.jp)

**Table S1 Cubic model of calcium aluminium oxide Ca6Al7O16.**

| Atom | site | *x* | *y* | *z* | O | *U*iso(A˚ 2) |
| --- | --- | --- | --- | --- | --- | --- |
| Ca1 | 24*d* | 0.109230 | 0.00000 | 0.2525 | 0.8572 | 0.00804 |
| Al1 | 24*d* | 0.231570 | 0.231570 | 0.231570 | 1 | 0.00925 |
| Al2 | 12*b* | 0.37500 | 0.000000 | 0.254040 | 1 | 0.0050533 |
| O1 | 16*c* | 0.064440 | 0.064430 | 0.064430 | 1 | 0.01105 |
| O2 | 48*e* | 0.099800 | 0.192280 | 0.286760 | 1 | 0.0123867 |
| Ca2 | 48*e* | 0.059700 | 0.0 | 0.25000 | 0.1428 | 0.0164 |
| O3 | 12*a* | 0.130000 | 0.472800 | 0.248400 | 0.1428 | 0.03 |

**Table S2 Rietveld refinement results of as-sintered Ca6Al7O16.**

| Atom | site | *g* | *x* | *y* | *z* | *U*iso(A˚ 2)a |
| --- | --- | --- | --- | --- | --- | --- |
| Ca1 | 24*d* | 0.899(4) | 0.106(2) | 0 | 1/4 | 0.0152(4) |
| Ca2 | 24*d* | 0.101(3) | 0.059(1) | 0 | 1/4 | 0.005(3) |
| Al1 | 16*c* | 1.00(6) | 0.0185 | 0.0185 | 0.0185(1) | 0.0063(5) |
| Al2 | 12*b* | 0.949(6) | 3/8 | 0 | 1/4 | 0.0028(6) |
| O1 | 16*c* | 0.962(4) | 0.1855 | 0.1855 | 0.1855(1) | 0.0101(4) |
| O2 | 48*e* | 0.961(4) | 0.03639(9) | 0.4421(1) | 0.15047(8) | 0.0101(1) |
| O3 | 12*a* | 0.313(2) | 1/8 | 1/2 | 1/4 | 0.049(1) |
| H1 | 48*e* | 0.080(6) | 1/8 | 1/2 | 1/3 | 0.22(1) |

Space group: *I* 43*d* (No. 220), *a* = *b* = *c* = 11.97978(4) A˚ , *α* = *β* = *γ* = 90*◦*

*R*wp = 5.97, *R*p = 5.23, *S* = *R*wp/*R*e = 1.56, *R*B = 2.71, *RF* = 7.26.

*a*: Equivalent isotropic atomic displacement parameter.

**Table S3 Rietveld refinement results of H2 gas-treated Ca6Al7O16**.

| Atom | site | *g* | *x* | *y* | *z* | *U*iso(A˚ 2) |
| --- | --- | --- | --- | --- | --- | --- |
| Ca1 | 24*d* | 0.899(3) | 0.143(2) | 0 | 1/4 | 0.0149(4) |
| Ca2 | 24*d* | 0.101(3) | 0.191(2) | 0 | 1/4 | 0.006(6) |
| Al1 | 16*c* | 1.00(7) | 0.0184 | 0.0184 | 0.0184(2) | 0.0076(1) |
| Al2 | 12*b* | 0.949(5) | 7/8 | 0 | 1/4 | 0.0035(5) |
| O1 | 16*c* | 0.962(3) | 0.1855 | 0.1855 | 0.1855(1) | 0.0122(3) |
| O2 | 48*e* | 0.961(4) | 0.0364(1) | 0.4423(1) | 0.1505(1) | 0.0106(5) |
| O3 | 12*a* | 0.150(1) | 3/8 | 0 | 1/4 | 0.050(5) |
| H1 | 48*e* | 0.038(6) | 0 | 0.17 | 3/8 | 0.22(1) |
| H2 | 12*a* | 0.183(6) | 3/8 | 0 | 1/4 | 0.05(4) |

Space group *I* 43*d* (No. 220), *a* = *b* = *c* = 11.97885(2) A˚ , *α* = *β* = *γ* = 90*◦*

*R*wp = 6.23, *R*p = 5.45, *S* = *R*wp/*R*e = 1.82, *R*B = 2.90, *RF* = 4.72.
